# An improved allele-specific PCR primer design method for SNP marker analysis and its application

**DOI:** 10.1186/1746-4811-8-34

**Published:** 2012-08-24

**Authors:** Jing Liu, Shunmou Huang, Meiyu Sun, Shengyi Liu, Yumei Liu, Wanxing Wang, Xiurong Zhang, Hanzhong Wang, Wei Hua

**Affiliations:** 1 Key Laboratory of Biology and Genetic Improvement of Oil Crops, Ministry of Agriculture, Oil Crops Research Institute of the Chinese Academy of Agricultural Sciences, Wuhan, 430062, People’s Republic of China; 2 Institute of Vegetables and Flowers of the Chinese Academy of Agricultural Sciences, Beijing, 100081, People’s Republic of China

**Keywords:** SNP, AS-PCR, Mismatch, Polymorphism, Destabilization

## Abstract

**Background:**

Although Single Nucleotide Polymorphism (SNP) marker is an invaluable tool for positional cloning, association study and evolutionary analysis, low SNP detection efficiency by Allele-Specific PCR (AS-PCR) still restricts its application as molecular marker like other markers such as Simple Sequence Repeat (SSR). To overcome this problem, primers with a single nucleotide artificial mismatch introduced within the three bases closest to the 3’end (SNP site) have been used in AS-PCR. However, for one SNP site, nine possible mismatches can be generated among the three bases and how to select the right one to increase primer specificity is still a challenge.

**Results:**

In this study, different from the previous reports which used a limited quantity of primers randomly (several or dozen pairs), we systematically investigated the effects of mismatch base pairs, mismatch sites and SNP types on primer specificity with 2071 primer pairs, which were designed based on SNPs from *Brassica oleracea* 01-88 and 02-12. According to the statistical results, we (1) found that the primers designed with SNP (A/T), in which the mismatch (CA) in the 3^rd^ nucleotide from the 3’ end, had the highest allele-specificity (81.9%). This information could be used when designing primers from a large quantity of SNP sites; (2) performed the primer design principle which forms the one and only best primer for every SNP type. This is never reported in previous studies. Additionally, we further identified its availability in rapeseed (*Brassica napus L.*) and sesame (*Sesamum indicum*). High polymorphism percent (75%) of the designed primers indicated it is a general method and can be applied in other species.

**Conclusion:**

The method provided in this study can generate primers more effectively for every SNP site compared to other AS-PCR primer design methods. The high allele-specific efficiency of the SNP primer allows the feasibility for low- to moderate- throughput SNP analyses and is much suitable for gene mapping, map-based cloning, and marker-assisted selection in crops.

## Introduction

Single Nucleotide Polymorphisms (SNPs) are single base differences between DNA of different individuals. Once discovered, SNPs can be converted into genetic markers that can be assayed [1,2]. As the most abundant and stabile form of genetic variation in most organism genomes, SNPs are more suitable for genotyping markers compared to the conventional markers such as RFLP (Restriction fragment length polymorphism), AFLP (Amplified fragment length polymorphism) and SSR (Simple Sequence Repeat). With the development of bio-technology, SNPs are becoming favored genetic markers that are used in marker-assisted breeding [3], map-based cloning [4], study of evolutionary conservations between different species [5,6], and the detection of risk-associated alleles linked to human diseases [7].

Recently, massive parallel sequencing platforms such as GSFLX (Roche), Solexa (Illumina) and SOLID (Applied Bios stems) have significantly reduced the cost of high throughout sequencing [8]. A large number of genomes and transcriptomes have been rapidly sequenced using these new platforms to identify novel SNPs in maize [9], rapeseed [10] and human [11] etc. A large variety of techniques for high-throughput SNP genotyping have also been developed using Taqman [12], Amplifluor [13], genome re-sequencing [14,15], and SNP arrays [16,17]. These techniques are expensive and require specialized equipments, which cost more standard primers and are not practical for assaying low- to moderate-throughput SNPs. Hence, there is a need for simple and accurate genotyping assays that can be implemented in laboratories lacking access to sophisticated equipment.

Traditional SNP genotyping methods such as CAPs (The Cleaved Amplified Polymorphic Sequence), dCAPs (derived CAPS), and AS-PCR (Allele-specific PCR) are widely used for low-throughput applications in plant research. In application, CAPS and dCAPS are restricted by end nuclease sites that could be inefficient and not cost-effective [18-20]. AS-PCR is based on the extension of primer only when its 3’end is a perfectly complemented to the template [21]. In principle, SNPs can be detected using allele-specific PCR primers based on the 3’ terminal nucleotide of a primer that corresponds to a specific SNP site. However, reliable discrimination between the alleles is not sufficient to achieve using this described method. To overcome this problem, allele-specific primers with an additional base pair change within the three bases closest to the SNP site between alleles have been used [21,22]. Each specific SNP site in an allele can generate at least 18 possible primers with one mismatch base [23]. The SNAPER program generates a list of up to 16 possible primers per SNP site for each allele [23]. Therefore, choosing additional mismatches to increase primer specificity has been a challenge for AS-PCR [23]. Some studies have proposed criteria for designing AS-PCR primers. Hayashi *et al* (2004) proposed that base pair mismatches created through T-G or C-A transversions at third base from 3’ end could increase the allele-specificity [24]. Hirotsu *et al* (2010) identified A-T transversion and A-G transition were useful base pair mismatches for improvement of allele-specific amplification [25]. The WASP tool could also be used to introduce mismatches at the penultimate (2^nd^ to the terminal) base of the primer [26,27]. However, most studies used only a limited quantity of primers, which might have some influences on efficiency of SNP primer specificity.

In this study, over 2000 primer pairs, which were designed based on SNPs between *B. oleracea* lines 01-88 and 02-12, were used to analyze the effects of different SNP types, mismatch bases and sites within the three bases closest to the 3’end on primer specificity. Based on these results, we advanced the SNP primer design principle. Compared to traditional SNP genotyping methods, our method could provide a cost-effective alternative for high efficient specific primers and would greatly facilitate plant research.

## Results

### SNP analysis of *B.oleracea* 01-88 and 02-12 genome sequences

The assembly of genome sequences of *B.oleracea* line 02-12 has been accomplished (unpublished). To identify SNPs between *B. oleracea* lines 01-88 and 02-12, genome DNA of line 01-88 was re-sequenced and a total of 119 million reads were obtained. To get high-quality SNPs, the sequence data was subjected to stringent filtering: The reads from line 01-88 were compared to the sequences of line 02-12 using BLASTN. Sites containing tri-allelic or high degree of polymorphism were omitted. The sequences containing over 8 reads mapped to unique sites in the genome sequence were extracted from line 01-88. Pair wise alignment was used to evaluate the SNPs between genome sequences of 01-88 and 02-12. The alignment result revealed a total of 1,422,113 SNPs existed between *B. oleracea* lines 01-88 and 02-12 (an average of one SNP in every 360 bp fragment) (Table 1). Analysis to the SNPs showed over half (56.7%, 806387/1422113) of the nucleotide changes were transitions (A-G or C-T). Transversions (A-T, A-C, C-G and G-T) accounted for 43.3% (615726/1422113) of the detected SNPs (Table 1).

**Table 1:** **Putative SNPs identified between *B. oleracea *genomes of 01-88 and 02-12**

**SNP (01-88/02-12)**	**No. of every SNP type**	**Caused by**	**Total No. of transition and transversion**
**A/G**	**201247**	**transition**	**806387**
**C/T**	**202316**		
**G/A**	**202801**	**transition**	
**T/C**	**200023**		
**A/C**	**79813**	**transversion**	**615726**
**G/T**	**79800**		
**C/A**	**80029**	**transversion**	
**T/G**	**79782**		
**A/T**	**99848**	**transversion**	
**T/A**	**99743**		
**G/C**	**48372**	**transversion**	
**C/G**	**48339**		

To further identify the putative SNPs and estimate the proportion of false positives, 96 SNP sites derived from only 8-read sequences were chosen randomly. Primers were designed according to the genome sequences near these SNP sites and all amplicons could generate about 500 bp fragments in which containing the corresponding SNPs. Sanger sequencing results showed 93 SNPs were identical with the putative SNPs and 3 SNPs were unpredicted or undetectable. This indicated a very high proportion of SNPs really existed between lines 01-88 and 02-12.

### Design and efficiency detection of SNP primers

**Figure 1:**
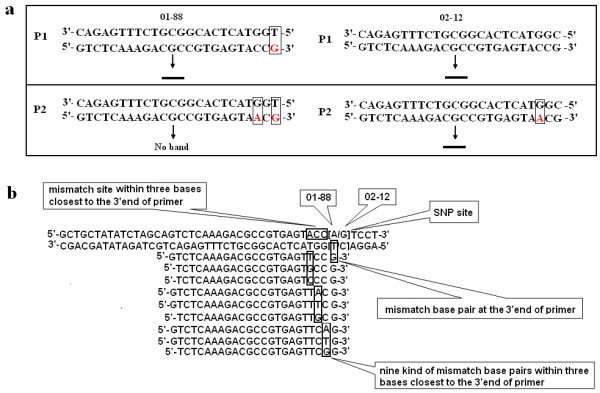
**Schematic representation of the AS-PCR primer design.a**, Primer P1 forms a perfect match with allele from 02-12, but a mismatch base pair at the 3’end with the DNA sequence of allele from 01-88. It could amplify the band in both of two lines 01-88 and 02-12. Primer P2 forms two mismatch base pairs with allele from 01-88 at the 3’end and in the 3^rd^ nucleotide from the 3’end, while a mismatch base pair in the 3^rd^ nucleotide with allele from 02-12. It amplified the band only in 02-12. **b**, Schematic representation of different mismatches during the SNP primer design.

Primer design strategy for allele-specific PCR was illustrated in Figure 1. Primer P1, which was designed based on 02-12 genome sequence, formed a mismatch (TG) in 3’end with the DNA sequence of 01-88. While in most cases, it still could amplify the band with 01-88 efficiently. After introducing another mismatch (GA) in the 2^nd^ site closest to the 3’end in primer P2, it could only amplify the band with 02-12 (Figure 1a). Based on the method described by Cha *et al* (1992), mismatch sites and mismatch bases closest to the SNP site were randomly chosen [21]. Therefore, nine possible mismatches could be generated among the three bases closest to 3’end of primer (Figure 1b).

In this study, 1686 *B. oleracea* SNPs including 12 kinds of SNPs were chosen for primer design ( Additional file 1). Among them, some of those SNPs could form the same 3’end mismatches in 01-88 with allele-specific primers. Therefore, all SNPs could be classified into eight kinds including A/G and C/T, T/C and G/A, A/C and G/T, T/G and C/A, A/T, T/A, C/G, G/C (Table 2) based on mismatch types (for example, for SNPs A/G and C/T, the 3’ end mismatch of the allele-specific primer designed for 02-12 is TG in 01-88). Moreover, for convenience of data analysis, we further compressed into four SNP types including A/G (T/C), A/C (T/G), A/T (T/A) and G/C (C/G) based on their destabilization effects in mismatch base pairs (Table 2) [28,29].

**Table 2:** Destabilization strength of eight combinations of mismatch nucleotide pairing

**SNP types**	**mismatch types at 3’end of primer in 01-88**	**Destabilization strength of mismatch type**
**(01-88/02-12)**		
**A/G**	**TG**	**Strong**
**C/T**	**GT**	
**G/A**	**AC**	
**T/C**	**CA**	
**A/C**	**TC**	**Weak**
**G/T**	**CT**	
**C/A**	**AG**	
**T/G**	**GA**	
**A/T**	**TT**	**Strong and Medium**
**T/A**	**AA**	
**G/C**	**CC**	**Strong and Medium**
**C/G**	**GG**	

In this study, all the primers were used to amplify the genome DNA of lines 01-88 and 02-12, and PCR products were detected on 2.5% agarose gel by electrophoresis. The results showed that among the 1686 primer pairs, 490 pairs (29.1%) displayed polymorphism (Table 3). When classified by SNP types, the percents of polymorphism of A/G (C/T), A/C (G/T), A/T (T/A), C/G (G/C) were 26.4% (111/420), 31.3% (131/418), 34% (148/435) and 37.5% (155/413) respectively. When classified by mismatch sites, polymorphism percents of the 2^nd^, 3^rd^ and 4^th^ base location closest to the SNP sites were 25.1% (139/554), 32.9% (189/575) and 29.1% (162/557), respectively. If SNP type and mismatch site in primers were both considered, the polymorphism percent of SNP A/T (T/A) which had mismatches in the 3^rd^ site was 45.9% (72/157), followed by C/G (G/C) in the 2^nd^ site with 43.3% (58/134) and 3^rd^ site with 39.4% (56/142). The polymorphism percent of SNP A/G (C/T) was 26.4% (111/420), which also has the lowest polymorphisms in all mismatch sites.

**Table 3:** Effect of mismatch sites and SNP types on the specificity of allele-specific PCR

**Mismatch sites closest to the 3’end of primers**	**Polymorphism percent of SNP primers**	**Polymorphism percent of primers for every SNP type**			
		**A/G, C/T**	**A/C, G/T**	**A/T, T/A**	**C/G, G/C**
**The 2**^**nd**^**base**	**25.1%**	**23.0%**	**26.8%**	**26.6%**	**43.3%**
	**(139/554)**	**(32/139)**	**(37/138)**	**(38/143)**	**(58/134)**
**The 3**^**rd**^**base**	**32.9%**	**30.7%**	**37.4%**	**45.9%**	**39.4%**
	**(189/575)**	**(42/137)**	**(52/139)**	**(72/157)**	**(56/142)**
**The 4**^**th**^**base**	**29.1%**	**25.7%**	**29.8%**	**28.1%**	**29.9%**
	**(162/557)**	**(37/144)**	**(42/141)**	**(38/135)**	**(41/137)**
**Total number**	**29.1%**	**26.4%**	**31.3%**	**34%**	**37.5%**
	**(490/1686)**	**(111/420)**	**(131/418)**	**(148/435)**	**(155/413)**

Moreover, the polymorphism percents of SNP primers classified by the types of mismatch bases in the 2^nd^, 3^rd^ and 4^th^ mismatch sites were analyzed (Table 4). Results showed that polymorphism percents of 8 mismatch types were closely equivalent in the 2^nd^ sites (22.5%-26.4%). High polymorphism percents appeared in two mismatch base pairs in the 3^rd^ base location (CA, 46.8%, 37/79; TG, 38.6%, 27/70). At the 4^th^ base away from the SNP site, the polymorphism percents of primers with mismatch base pairs GA, TC, TT and CC were over 30% (31.7%-33.3%).

**Table 4:** Effect of artificial base mismatches in three mismatch sites on the specificity of allele-specific PCR

**Mismatch sites closest to the 3’end of primers**	**Mismatch types**	**Polymorphism percent of primers**	**Mismatch types**	**Polymorphism percent of primers**	**Mismatch types**	**Polymorphism percent of primers**	**Mismatch types**	**Polymorphism percent of primers**
**The 2**^**nd**^**base**	**TC**	**26.2%**	**GA**	**22.5%**	**CA**	**25.7%**	**AA**	**23.8%**
		**(17/65)**		**(16/71)**		**(19/74)**		**(15/63)**
	**TG**	**26.4%**	**GG**	**24.2%**	**CC**	**26.1%**	**TT**	**25.6%**
		**(19/72)**		**(15/62)**		**(18/69)**		**(20/78)**
**The 3**^**rd**^**base**	**TC**	**32.1%**	**GA**	**28.3%**	**CA**	**46.8%**	**AA**	**30.6%**
		**(26/81)**		**(17/60)**		**(37/79)**		**(26/85)**
	**TG**	**38.6%**	**GG**	**30.4%**	**CC**	**23.9%**	**TT**	**29.7%**
		**(27/70)**		**(21/69)**		**(16/67)**		**(19/64)**
**The 4**^**th**^**base**	**TC**	**32.9%**	**GA**	**31.7%**	**CA**	**28.0%**	**AA**	**26.8%**
		**(24/73)**		**(26/82)**		**(21/75)**		**(19/71)**
	**TG**	**23.3%**	**GG**	**25.8%**	**CC**	**33.3%**	**TT**	**32.4%**
		**(14/60)**		**(16/62)**		**(22/66)**		**(22/68)**

**Figure 2:**
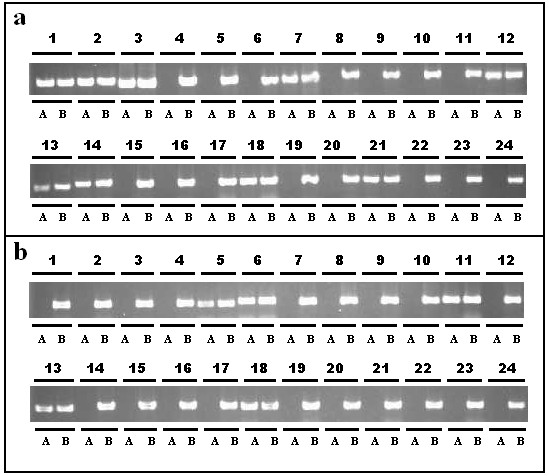
**Analysis of specificity for SNP primers of *B. oleracea*.a**, 1-24 primer pairs, which corresponded to Bo001-Bo024, were introduced with a CA base pair mismatch in the 3^rd^ nucleotide closest to 3’end (A/T SNP type, AA mismatch). **b**, 24 primer pairs, which corresponded to Bo194-Bo217, were introduced with a CA base pair mismatch in the 3^rd^ nucleotide closest to 3’end (A/T SNP types, TT mismatch). A, 01-88; B, 02-12.

Based on the results in Table 3 and Table 4, we inferred that primers, which had CA mismatch in the 3^rd^ base closest to SNP site A/T (T/A), had the highest polymorphism between lines 01-88 and 02-12. To identify this conjecture, 385 primers designed from SNP A/T (T/A), which contained G or T base in the 3^rd^ nucleotide, were further chosen for polymorphism analysis ( Additional file 2). The detection results showed that 295 SNP primers were polymorphic (295/385, 76.6%) between lines 01-88 and 02-12. When these primers were classified based on the SNPs (A/T or T/A) in 3’end, the specificity percent of SNP (A/T) (158/193, 81.9%) was higher than that of SNP (T/A) (137/192, 71.4%) (Figure 2a and Figure 2b).

### Application of the design method

Besides the highest polymorphic primers mentioned above, the high polymorphic primers could be found in every SNP type based on our results. The principle of primer design is described as followed: firstly, for every kind of SNP, the mismatch site (the 2^nd^, 3^rd^ and 4^th^ site closest to the SNP site) is chosen according to the result of polymorphism percent in Table 3. For the primers of three SNP types A/T (T/A), A/C (G/T) and A/G (C/T), the highest polymorphism percents are 45.9%, 37.4% and 30.7%, respectively and the mismatches in primers are all located in the 3^rd^ site. While for the SNP type C/G (G/C), the mismatches in the 2^nd^ site closest to 3’end of primers show highest polymorphism percent (43.3%). For the base in mismatch site (the 2^nd^, 3^rd^ and 4^th^ site closest to the SNP site), there exist three different mismatch styles for every kind of base. The best mismatch style is chosen according to the statistical results from Table 4. For example, if there is a G in the 3^rd^ site, the mismatches of CC, CA and CT will be formed. The polymorphism percent of primers with CA (46.8%) mismatch is highest compared to that of CC (23.9%) and CT (32.1%).

**Figure 3:**
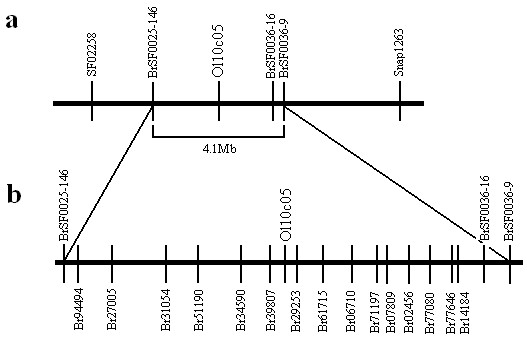
**SNP marker density increase of rapeseed linkage map.a**, An oil content QTL was scanned between O110C05 and BrSF000036-9. **b**, Among twenty primers, fifteen new makers were generated for further QTL location.

We further identified the usability of the primer design method on rapeseed and sesame. During rapeseed oil content research, with DH lines of F1 generation between zy036 and 51070, which had been reported by Hua *et al*[30], we identified a QTL related to oil content located on A2 chromosome. Because of lack of markers in the QTL interval, we designed a total of 20 SNP primers between marker O110C05 and marker BrSF000036-9 using our method (Table 5). Results showed that 15 primers were located in the QTL interval (Figure 3). The polymorphism percent of primers is (15/20, 75%). Another 24 primers came from A/T SNPs between two sesame lines 28-31 and ZZM2289 were also designed. And among them, 18 SNP markers were detected to have polymorphism (18/24, 75%) ( Additional file 3). Both results from rapeseed and sesame indicated that the SNP primer method could be used in other species.

**Table 5:** 20 primers pairs designed according to SNPs between rapeseed zy036 and 51070

**Primer name**	**SNP type**	**Mismatch site closest to 3’end of primers**	**Mismatch base pairs**	**Forward primer(5’-3’)**	**Reverse primer(5’-3’)**
Br94494	C/G	2^nd^	CT	AGTTACATAGGTCCACAATCATAGAATAAACTTTTC	TACCATTCGGAGCCTAAATAGAGGTAAAAGGTG
Br27005	T/A	3^rd^	CA	AAGATTGTTTCAAACGCAAAAATATACAACAAAAT	CAACGAATTTCACACTTTAGTAATGCACTGAGATTT
Br31054	T/A	3^rd^	CA	CAAGTGAGACTGAATCCACAATAAAGGATGCTACT	TCGGATAAAATCCCCAGCTCTACTATACATTCC
Br43193	T/A	3^rd^	CA	CCTTTTATTTGATCACAGGGGTTTGTAGGAACT	TCAGCCAGTAACGTCCCCCACATC
Br51190	T/A	3^rd^	CA	AAATAATGGCATGCTCCTCTTTAATCTACCAAACT	TATTCGGTTCCGAAAATAATGCGATGC
Br34590	T/A	3^rd^	CA	AAGTGACGGTTCTTTAAGTTATCAGAGTCTCCTAAT	AGATTTGGGATTAAAATCAAGTTGTGGGTTAGTTTT
Br03637	T/A	3^rd^	CA	ATTACAGAATGTGTGTGCAAACAGAAATACATTACT	TGTGTCCCCATTTCGTGTAATCATAAAGCTAG
Br39807	T/A	3^rd^	CA	CGAGACTCGGGTCGTTGAGTGGAAAT	ATCCTAAAGACTTCTCCCACAAATCCACCAT
Br68275	T/A	3^rd^	CA	TGCCGCATGTATGTCGGAGATGATAAT	AACCGAAACCCTAGTAGGCTAGGCGC
Br29253	T/A	3^rd^	CA	TGGCGCTAAATCCAAGAAGAAGTCCATT	AATTACCACCTTTCTTACCCTTGTTACTCATGACAG
Br61715	T/A	3^rd^	CA	TGAATAGATTCTTCCGCATCACCTTTTAAAGTTAAT	CTTGTTTCAAGAGAAATTGAACAAGCTGCAGT
Br24494	C/G	2^nd^	GT	GTCAATAATACTAGCAAACATACAACAGCGAGATTC	TTGCAAATTTTAGTCAAAGTCGGTAGAAAATAGATC
Br06710	T/A	3^rd^	CA	TCTTGTCGATGCTGAGCTGGCAAATACT	GGTCAAGCTCACACACACTCCACGTC
Br00855	T/A	3^rd^	CA	CACTATGGGCTATGGTGGGTCCTTCAAT	TGATTGGAGTTCTGTGCTCGTAGTTTTGC
Br71197	C/G	2^nd^	GT	TATGGCACACAGACAGAGTTCCAGGAAATC	TCTTCCAGTTCGATATCTTGGTCTGTCCC
Br07809	C/G	2^nd^	GT	CTCCGCCCACATGTTATAATATGTCAGTATATCTTC	TAGTGAATGGAGAAAGAGAACAAAGCCTACAGTACA
Br02456	T/A	3^rd^	CA	GCCTTCAGAAGGTCTGGAAACTGGATT	GTTCGATGGACTTCACTACCTCCCATAGCT
Br77080	T/A	3^rd^	CA	CGGATAGTTTCGGGTTCGGTTCGATT	ACCGAACGGGTACCCGAATATATAAAAATATTAATT
Br77646	T/A	3^rd^	CA	TCCACCAGAATTGTGTGATGGCACTTACT	GAAAAACGTCAGGTCAATGTATCAACTTCGATAA
Br14184	G/A	3^rd^	AC	GATTAACCGATGAAAGTCTCAGTGCCACAG	TCGTCTGTGACTCCCAAACACTTGGATAG

## Discussion

In recent years, various methods for high-throughput SNP analysis have been described [28]. Although these methods are highly efficient compared to other traditional SNP genotyping by electrophoresis, significant investments of expensive probes, microchips or special instrumentation have limited their use in most laboratories. For traditional low-throughput SNP genotyping methods, the main time and labor, and low efficiency of specific primer are still challenges [27]. The Allele-specific PCR method was developed for allele analysis of clinically significant mutations. To facilitate reliable discrimination between two alleles highly, the addition of artificial mismatches within the three bases from 3’end of the primers might be beneficial [23]. Although the third position from the 3’end has been detected as the best to place a mismatch base in primer previously [24], we really do not know which kind of mismatch (for every base, there are three kinds of mismatches) is the best choice in the 3^rd^ position. In this study, different from the previous reports which used only a limited quantity of primers, a large amount of SNP primers designed by introducing mismatches within the three bases closest to the 3’end of primers were used to solve this problem.

Generally, AS-PCR primers designed randomly had a low allelic specificity rate of approximately 30%, which was consistent with our results (29.1%). However, mismatch sites (2^nd^, 3^rd^, and 4^th^ site closest to the 3’end) had different effects on the polymorphic efficiency of primers. In our study, we found primer polymorphic percent was lowest in the 2^nd^ base location because many primers could not amplify any bands in both of the lines 01-88 and 02-12. For the 4^th^ base location, the polymorphism efficiencies of all mismatch types were almost equivalent (under 30%). The highest polymorphic percent was found in the 3^rd^ base located closest to the SNP site, which was observed by Hayashi et al similarly (2004) [24].

According to the results of thermodynamics of mismatches reported by Peyret *et al* (1999) and Little (2001) [28,29], the mismatch base pairs had different destabilization effects that could be divided into weak, medium, and strong strength of destabilization. Therefore, during design of AS-PCR primers, the effects of mismatches no matter in 3’end or within the three bases closest to the 3’end of primers should be both considered [26,27]. In this study, for convenient analysis, we compressed four SNP types including A/T (T/A), A/G (T/C), A/C (T/G), and G/C (C/G) based on their destabilization effects of mismatch base pairs formed in 3’end of primers. Among them, primers generated from SNP types A/G (T/C) had the lowest detection efficiency in all mismatch sites. It was reasonable because AC and GT mismatches had weak destabilization strength. The primers including these specific mismatches at 3’end were easier to make amplification in both alleles.

Similarly, mismatch types within the three bases closest to the 3’end affect specificities of primers. In the 3^rd^ base, CA and TG (the highest polymorphic mismatches) belonged to weak destabilization strength mismatches. The mismatches GA, TC, TT, and CC (the higher polymorphic mismatches) located at the 4^th^ base away from the SNP site belonged to the strong destabilization strength mismatches. From the results, we deduced that SNPs (A/T), which contained CA mismatches in the 3^rd^ nucleotide from the 3’ end of the primers, had the highest allele-specification. According to the combination rules, polymorphic efficiency between TT (mismatch in 3’end of primer, strong destabilization strength) and CA (weak destabilization strength) are typically higher than AA (mismatch in 3’end of primer, medium destabilization strength) and CA. Our results confirmed this deduction.

Based on these results, we performed the primer design principle which could form the one and only best primer for every SNP type. Among them, mismatches in the second positions were more appropriate for SNP type (C/G and G/C), which was different from the viewpoint that mismatch in the 3^rd^ position was the best choice for AS-PCR. With the primer design principle, we further tested the primers designed based on SNPs of rapeseed and sesame. High efficient polymorphism of the primers identified the usability of the method in other species.

## Conclusion

A SNP primer design method was developed which improved the polymorphism efficiency of AS-PCR primers highly. The modified primer design can help to identify the best effective primer for each SNP and potentially is a valuable tool for gene mapping, map-based cloning and marker-assisted selection in crops.

## Methods

### Plant materials and SNP information

At least 20ug genome DNA of *B. oleracea* lines 01-88 and 02-12 at a concentration of ≥50 ng/ul, was sent for Solexa sequencing as a commercial service. The DNA was fragmented into small pieces using divalent cations at elevated temperature. The cleaved short DNA fragments were prepared for Solexa sequencing in BGI (China). REPEAT MASTER was used for screening repeated sequences with default parameter and labeling the sequences from different materials. For genome location of fragments, SOAP adapting the default parameter values was used for the initial alignment and screening to avoid the effects of paralog [31]. SNP primer design was performed using screened results.

### SNP analysis and verification by Sanger sequencing

To verify the putative SNPs, 96 SNP sites derived from only 8-read sequences were randomly chosen between *B. oleracea* lines 01-88 and 02-12. Primers (Sangon, China) were designed to amplify about 500 bp fragments in which containing the corresponding SNPs. The PCR reaction contained 25 ng DNA, 0.2 mM dNTP, 0.5U Taq (MBI, USA) with 1xbuffer, and 5pM of each primer. PCR parameters were as follows: a pre-denaturation of 94°C for 2 min, 35 cycles of amplification (94°C for 30S, 60°C for 1 min and 72°C for 1 min) and a final extension reaction was performed at 72°C for 5 min. PCR products were detected on 1.0% agarose gel by electrophoresis and ligated into PMD18T-vector (Takara, Japan) for SNP identification.

### Primer design and testing

Allele-specific primers corresponding to 12 kinds of SNP in *B. oleracea* were designed according to different combinations between mismatch base and mismatch site. Optimization of melting temperature, primer length and amplified products length were achieved using primer program WebSNAPER ( http://pga.mgh.harvard.edu/cgi-bin/snap3/websnaper3.cgi) [23]. Primer sequences were screened against *B. oleracea* genome repetitive sequences to minimize mis-priming.

Polymorphism assay of SNP primers were performed by PCR and detected by agarose gel electrophoresis. All the forward primers are allele-specific for *B. oleracea* line 02-12 and the reverse primer is not allele-specific. Amplification of SNP primers was performed on C1000TM Thermal Cycler (Bio-Rad, USA) using 20 ul reactions. Before carrying out this study, we had chosen some Taq polymerases: MBI Taq DNA Polymerase and Takara Taq (two general Taq), MBI Dream Taq^TM^ DNA Polymerase and Takara Ex Taq (which are better in amplification efficiency and sensitivity compared to general Taqs) to identify their effects on amplification efficiency. Result showed both of general polymerases (MBI Taq DNA Polymerase and Takara Taq) had same and high allele-specific amplification efficiency compared to the other two Taqs. Therefore, general Taq polymerase would be best choice in allele-specific PCR and Taq DNA Polymerase from MBI was chose in this study. The PCR reaction contained 25 ng DNA, 0.2 mM dNTP, 0.5U Taq DNA Polymerase with 1xbuffer, and 5 pM of each primer. PCR parameters were as follows: a pre-denaturation of 94°C for 2 min, 35 cycles of amplification (94°C for 30s, 55°C-65°C for 1 min and 72°C for 30s) and a final extension reaction was performed at 72°C for 10 min. PCR products were separated on 2.5% agarose gel by electrophoresis.

### Application in rapeseed and sesame

Rapeseed DNA samples including two parents (high oil content line zy036 and low oil content line 51070) and DH lines, which had been reported by Hua *et al*[30], were prepared using the DNAeasy plant kit miniprep (Qiagen, Valencia, CA). Zy036 and 51070 were re-sequenced and blasted with *B. napus* genome sequence (unpublished). Additionally, two sesame lines 28-31 and ZZM2289 (genome sequence has not been published) were also used in our research. All SNPs were chosen according to the method described in *B. oleracea* lines 01-88 and 02-12. The SNP primers were designed according to our primer design method.

## Abbreviations

SNP: Single Nucleotide Polymorphism; AS-PCR: Allele-Specific PCR; SSR: Simple Sequence Repeat; RFLP: Restriction fragment length polymorphism; AFLP: Amplified fragment length polymorphism; CAPs: The Cleaved Amplified Polymorphic Sequence; dCAPs: Derived The Cleaved Amplified Polymorphic Sequence.

## Competing interests

The authors declare that they have no competing interests.

## Authors’ contributions

JL and SMH contributed to primer design and took the co-lead role in writing the manuscript. SMH and MYS performed the primer analysis. SYL offered sequence data of *B. oleracea and B. napus*. YML and WWX provided the DNA of *B. oleracea* 01-88 and 02-12. XRZ provided the genome sequence and DNA of *S. indicum L*. 28-31 and ZZM2289. HZW participated in discussions during experimental work. WH conceived the project and approved the final version of the manuscript. All authors read and approved the final manuscript.

## Supplementary Material

Additional file 1 **1686 primer pairs designed based on the SNPs between*B. oleracea*lines 01-88 and 02-12.** Click here for file

Additional file 2 **385 primer pairs designed based on the SNPs between*B. oleracea*lines 01-88 and 02-12 which are A/T SNP type with G and T base in the 3**^**rd**^**nucleotide.** Click here for file

Additional file 3 24 primer pairs designed based on the SNPs between sesame lines 28-31 and ZZM2289. Click here for file
